# Copper Metabolism in Isolated Macrophages: Regulator of Immunity and Inflammation

**DOI:** 10.3390/vetsci13060511

**Published:** 2026-05-24

**Authors:** Xinao Leng, Ping Yu, Zhidi Xu, Chenglong Xia, Rui Du, Qiwen Luo, Yanqiu Zhu, Hongrui Guo

**Affiliations:** 1 College of Veterinary Medicine, Sichuan Agricultural University, Chengdu 611130, China; lxa3219385737@outlook.com (X.L.); yuping@stu.sicau.edu.cn (P.Y.); 17835424879@163.com (Z.X.); 2023203022@stu.sicau.edu.cn (C.X.); durui1@stu.sicau.edu.cn (R.D.); 2024303130@stu.sicau.edu.cn (Q.L.); zhuyanqiu@sicau.edu.cn (Y.Z.); 2 Key Laboratory of Animal Diseases and Environmental Hazards of Sichuan Province, Sichuan Agriculture University, Wenjiang, Chengdu 611130, China

**Keywords:** macrophages, copper, antimicrobial activity, inflammatory response, tissue repair

## Abstract

Copper is essential for the proper functioning of immune cells and participates in diverse biochemical processes. The maintenance of copper ion homeostasis is critical for normal host physiology, while dysregulation of copper metabolism is closely linked to various diseases. Emerging evidence indicates that disease-associated elevations in copper levels significantly enhance macrophage functions, including the expression of inflammatory cytokines, phagocytosis, and bactericidal activity. As key innate immune cells, macrophages not only eliminate invading pathogens but also contribute to immune regulation, tissue repair, and angiogenesis. In this review, we summarize current knowledge of copper transport and homeostatic mechanisms in macrophages and highlight how copper regulates their antimicrobial activity, inflammatory responses, and reparative functions. A deeper understanding of these mechanisms may provide new insights into therapeutic strategies targeting macrophage regulation through copper metabolism in the context of infectious and inflammatory diseases.

## Simple Summary

Copper is an essential nutrient for humans and animals, and macrophages—key immune cells that fight off harmful germs and regulate the body’s immune responses—rely on balanced copper levels to work properly, yet how copper shapes macrophage function in fighting infections and reducing inflammation is not well understood by the public. This review aims to clearly explain how copper is moved and balanced inside macrophages, and how it affects these cells’ ability to kill bacteria, control inflammation, and help repair damaged body tissues. We found that copper boosts macrophages’ antibacterial activity by creating harmful molecules to destroy germs and moving into germ-containing cell compartments to trigger toxicity, and it also controls inflammatory signals in the body while aiding tissue repair by strengthening key structural proteins. We conclude that copper is a critical regulator of macrophage function. This knowledge helps the public and researchers understand copper’s role in immunity and paves the way for new treatments for infectious and inflammatory diseases, as well as better wound repair methods in medicine.

## 1. Introduction

Copper is one of the essential trace elements for humans and animals, closely related to important life activities such as hematopoiesis, growth, and reproduction [1]. Most of the copper in the blood exists in a combined form [2]. It is an important component of many enzymes in the body, such as cytochrome c oxidase (CCO), ceruloplasmin (CP), and superoxide dismutase (SOD), and is widely involved in processes like oxidative phosphorylation and detoxification of free radicals [3]. Copper indirectly affects iron absorption, promoting the synthesis of hemoglobin and heme in the bone marrow; therefore, the most common sign of copper deficiency is anemia [4]. In cases of severe copper deficiency in humans and animals, serum copper and CP levels decrease, and symptoms of reduced erythrocyte SOD activity appear [5]. Copper is involved in a variety of biochemical processes in cells, such as mitochondrial respiration [6], connective tissue formation, pigmentation, iron oxidation, neurotransmitter processing, and antioxidant defense [1]. The redox properties of copper allow it to participate in electron transport, but it can also lead to its toxicity [7]. As a result, cells have evolved copper-processing proteins to ensure that copper is safely transported to specific utilization sites [3].

As an important component of host immunity, macrophages play an important role in the body’s resistance to bacterial invasion. The study of the physiological and pathological processes of macrophages resisting bacteria is of great guiding significance for clinical diagnosis and treatment [8]. When a pathogen attacks the host, macrophages respond quickly and activate to eliminate bacterial and fungal pathogens and virus-infected host cells through phagocytosis, while secreting cytokines, chemokines, and other factors to awaken the immune system, such as antigen presentation [8].

Copper is essential for the proper functioning of immune cells and participates in diverse biochemical processes. Copper deficiency can lead to impaired immune function and increased susceptibility to infection [9]. In macrophages, copper ions promote cell activation and trigger inflammatory responses, highlighting their pivotal role in host defense. Recent studies not only underscore the importance of copper in host–pathogen interactions but also provide new insights into how nutritional immunity contributes to the control of intracellular pathogens. Moreover, these findings suggest that modulating copper availability may represent a promising therapeutic approach to strengthen host defenses against infection [8,9]. In this review, we summarize the mechanisms of copper transport and homeostasis in macrophages and discuss their roles in antimicrobial activity, inflammatory responses, and tissue repair. We also highlight the emerging significance of copper metabolism in innate immunity and immunometabolism. Copper ions exert multifaceted effects on macrophage immune functions, and future research should further elucidate their underlying mechanisms and explore their translational potential in clinical therapy.

## 2. Copper Transport and Homeostasis in Macrophages

The distribution and metabolism of copper ions in cells are tightly regulated by a network of proteins, including the copper importer CTR1, the chaperone ATOX1, and the storage protein metallothionein [10,11]. Proper copper homeostasis is essential for maintaining host cell physiology, whereas excess copper can be toxic and is associated with multiple diseases [12]. Another critical transporter, ATP7A, resides in the Golgi apparatus and delivers copper into the secretory pathway to support the activity of copper-dependent enzymes [13]. Under hypoxic conditions, macrophages adapt by upregulating CTR1 expression and copper uptake, while simultaneously enhancing ATP7A expression and trafficking [14]. Copper and zinc are both essential for immune function and microbial defense. Macrophages increase uptake of these metals upon infection and deliver them to phagosomes to exert toxic effects on pathogens.

ATP7A is abundantly expressed in the vasculature and regulates the expression and activity of extracellular superoxide dismutase (SOD) [15]. Evidence from ATP7A-deficient mice demonstrates that peritoneal macrophages accumulate markedly higher copper levels compared to controls, confirming the essential role of ATP7A in maintaining copper balance in macrophages [16]. Notably, ATP7A also transports copper to ceruloplasmin in the secretory pathway, thereby contributing to iron homeostasis, especially under hypoxic conditions [17]. During hypoxia, copper distribution shifts toward preferential supply to ATP7A, accompanied by reduced activity of SOD1 and CCO. In tumor-associated macrophages, ATP7A expression and redistribution are strongly induced under hypoxia, potentially linking copper metabolism to tumor progression and angiogenesis [18].

## 3. Copper Enhances Macrophage Phagocytosis and Antibacterial Activity

### 3.1. The Regulation of Antibacterial Function in Macrophages

Macrophages employ diverse mechanisms to establish antibacterial responses, including LC3-associated phagocytosis (LAP), metabolic reprogramming, antimicrobial metabolites, lipid droplets (LDS), guanylate-binding proteins (GBP), antimicrobial peptides, metal ion toxicity, nutrient deprivation, autophagy, and nitric oxide (NO) production [19]. Notably, autophagy—particularly xenophagy—enables macrophages to degrade intracellular pathogens. Xenophagy serves as a compensatory mechanism targeting pathogens that have escaped into the cytoplasm or damaged phagosomes. This process is initiated by the ULK1/FIP200/ATG13 complex and relies on autophagy adaptor proteins (e.g., p62, NDP52) to recognize ubiquitinated pathogen components, subsequently guiding LC3-II incorporation into the nascent double-membrane phagophore [19]. Macrophages utilize multiple strategies to clear pathogens, including LAP, metabolic reprogramming, autophagy, and reactive oxygen/nitrogen species (ROS/RNS) generation [20]. LAP facilitates phagosome–lysosome fusion and bacterial degradation. Activated by pattern recognition receptors (e.g., TLR2, Dectin-1) during phagocytosis, LAP centers on NADPH oxidase assembly at the phagosome membrane to produce ROS. Concurrently, the RUBICON protein acts as a molecular switch, stabilizing NADPH oxidase and synergizing with the VPS34 complex to generate PI3P, driving LC3-II insertion into the single-membrane LAPosome and promoting lysosomal fusion for pathogen degradation. Studies indicate that upstream metabolic events—such as calcium–calmodulin signaling (e.g., during *Aspergillus* infection) and lipid second messengers like ceramide and diacylglycerol (DAG) (observed in *Listeria* and *Salmonella* infections)—are critical for NADPH oxidase activation. Metabolic shifts, including elevated mitochondrial ROS (MRO) and accumulation of antimicrobial metabolites, further enhance bacterial killing. LAP enhances phagosome–lysosome fusion by recruiting LC3 to phagosomes, improving macrophage bactericidal capacity. This process depends on ROS generated by the NOX2 complex and is potentiated by IL-6 signaling [21].

Metabolic reprogramming also contributes to macrophage antimicrobial function. Toll-like receptor (TLR) signaling reprograms mitochondrial activity to boost mitochondrial ROS (mROS) production and recruit mitochondria to phagosomes [22]. Metabolites such as RL5P and succinate, generated via the pentose phosphate pathway (PPP) and tricarboxylic acid (TCA) cycle, possess antimicrobial properties. LDs in macrophages are able to store neutral lipids and serve as energy reserves [23]. Studies have demonstrated that lipid droplets (LDs) can be recruited to macrophages to directly kill bacteria. Antimicrobial proteins on LDs, such as histones and antimicrobial peptides (AMPs), contribute to bacterial eradication. LDs not only serve as energy reservoirs but also function as platforms for storing antimicrobial proteins. For instance, histones and peptides directly kill pathogens. Upon bacterial invasion, pathogen-associated molecular patterns (PAMPs) released by pathogens are recognized by pattern recognition receptors (PRRs) on macrophage surfaces, activating the macrophages and triggering LD recruitment to infection sites (e.g., around bacteria-containing phagosomes). During this process, lipid droplet-associated protein PLIN2 is upregulated. PLIN2 acts as a molecular hub, specifically aggregating immune proteins like histones and AMPs to form functional “LD-antimicrobial protein complexes.” These complexes then migrate directionally and target bacteria (or bacteria-containing phagosomes) via surface molecular interactions. Ultimately, histones disrupt bacterial membrane integrity, while AMPs inhibit bacterial metabolism, synergistically eliminating pathogens. This process confirms that LDs are not merely traditional energy storage organelles but critical antimicrobial platforms mediating immune responses, highlighting their central role in integrating cellular metabolism and immune systems [24]. Metabolic reprogramming of macrophages is also closely related to antimicrobial responses [25]. GBPs are a class of GTPases induced by IFN-γ that promote antimicrobial defense through a variety of mechanisms, including the release of bacteria from bacteria-containing vesicles into the cytoplasm and the direct disruption of bacterial membranes [26,27]. GBPs, induced by IFN-γ, enhance host defense by disrupting bacterial membranes and promoting bacterial release from vesicles.

### 3.2. Copper Modulates ROS-Mediated Antibacterial Function

Copper can modulate the antimicrobial function of macrophages, including promoting copper accumulation in phagosomes, thereby enhancing antimicrobial activity, and copper may limit the growth of pathogens by promoting the production of reactive oxygen species (ROS) and the export of iron [28]. Copper distribution and transport in macrophages are regulated by inflammatory cytokines such as IFN-γ and TNF-α [29]. Copper-induced hydroxyl radical production is proposed as a major killing mechanism. Experimental studies have demonstrated that copper supplementation significantly reduces *E. coli* survival in RAW264.7 macrophages, with effects dependent on ROS generation [30]. Macrophages utilize ATP7A to actively transport cytoplasmic Cu^+^ into phagosomes. During phagosome maturation, metabolic H_2_O_2_ reacts with transported Cu^+^ in a Fenton-like mechanism, decomposing into highly toxic hydroxyl radicals (·OH), Cu^2+^, and hydroxide ions (OH^−^) [31]. This redox cycling enables sustained catalytic generation of ROS (reactive oxygen species) centered on ·OH. These radicals disrupt bacterial membrane lipids, protein active sites, and nucleic acid chains, compromising bacterial structural integrity and metabolic functions [10]. Copper enhances macrophage antimicrobial activity by promoting ROS production. Experimental studies have shown that *Streptococcus pneumoniae* is more susceptible to reactive oxygen species (ROS) generated through this pathway due to the lack of an effective ROS detoxification system and copper efflux mechanisms (such as in CopA-deficient mutants), which ultimately leads to its engulfment by macrophages. Copper supplementation reduces bacterial survival within macrophages, with this effect dependent on ROS generation [10].

### 3.3. Copper Participation in Antibacterial Process by ATP7A

ATP7A, a copper transporter, plays a vital role in trafficking copper to phagosomes, which is essential for bactericidal activity. For intracellular bacteria, stimulation with IFN-γ or LPS induces macrophages to increase ATP7A content on phagosome membranes, promoting copper transport into phagosomes and maintaining elevated copper concentrations within these compartments. This “copper toxicity” mechanism facilitates bacterial clearance through oxidative damage and metabolic disruption. In contrast, for fungal pathogens, IFN-γ activation of macrophages downregulates ATP7A expression in alveolar macrophages, reducing copper transport to phagosomes. The resulting decrease in phagosomal copper concentration creates a “copper restriction” environment that deprives fungi of essential nutrients, thereby inhibiting their proliferation [32]. Silencing the ATP7A gene via RNA interference (RNAi) significantly impaired macrophage bactericidal capacity, whereas exogenous copper supplementation restored this function. Hypoxia upregulated copper transporter CTR1 expression (2.7-fold higher than normoxic controls in RAW264.7 cells at 96 h) and induced ATP7A protein levels (increasing beginning at 24–48 h). This hypoxia-driven adaptation prioritized copper transport into the secretory pathway, activating ceruloplasmin to maintain copper homeostasis through coordinated metabolic reprogramming [14]. In unstimulated macrophages, ATP7A is predominantly localized to the Golgi apparatus, where it facilitates copper transport to metalloenzymes within the secretory pathway to maintain basal copper homeostasis. Upon stimulation with IFN-γ or LPS, macrophages undergo immune defense-oriented copper metabolic reprogramming, characterized by ATP7A dissociation from the Golgi apparatus and its vesicle-mediated trafficking into cytoplasmic vesicles. These vesicles exhibit spatial association and partial overlap with phagosomes (or their matured phagolysosomal counterparts), establishing a structural foundation for ATP7A-mediated copper delivery into the phagosomal lumen. The intraphagosomal copper reacts with H_2_O_2_ generated by NADPH oxidase to exert bactericidal effects. Relevant experiments have further confirmed that (Table 1) ATP7A is a key transporter for copper-dependent bacterial killing in macrophages (silencing ATP7A significantly inhibits the ability to kill *E. coli*, and the high killing effect against copper-sensitive bacteria can be reversed by ATP7A knockdown). The regulation of ATP7A trafficking by IFN-γ/LPS represents an adaptive response of the host innate immunity to infection [33].

### 3.4. Host–Pathogen Copper Competition

Copper ions can act both as antimicrobial toxic substances in host defense and as a defense against pathogens by limiting the availability of copper ions [34]. The host fights off infection by restricting pathogens’ access to essential nutrients such as iron, zinc, and copper, a mechanism known as “nutritional immunity” [35]. Macrophages inhibit bacterial growth by limiting the availability of nutrients such as iron, zinc, arginine, and tryptophan that are needed for bacterial growth. For example, NRAMP1/SLC11A1 restricts bacterial growth by removing magnesium from bacteria-containing phagosomes [36]. During infection with *Histoplasma capsulatum*, an ascomycete, facultative intracellular, thermally dimorphic fungus, the pathogen exists as a filamentous mold in the natural environment. Upon invading a host, it converts to a yeast form within alveolar macrophages, where it proliferates. Following initial pulmonary infection, alveolar macrophages phagocytose the fungus, which then germinates into yeast cells. Subsequently, infected macrophages can disseminate via the bloodstream to organs of the mononuclear phagocyte system, such as the liver, spleen, and bone marrow. In host defense, cellular immunity plays a dominant role: cytokines such as interferon-γ (IFN-γ) and TNF-α, secreted by CD4^+^ Th1 cells, activate macrophages and promote granuloma formation, thereby restricting pathogen proliferation [37]. Studies have shown that IFN-γ significantly reduces copper availability within the phagosome, forcing *H. capsulatum* to rely on its high-affinity copper transporter Ctr3 to acquire copper for survival. Moreover, Ctr3 expression is upregulated under copper-limited conditions and is essential for intracellular survival [32]. This illustrates how macrophages dynamically regulate copper to balance microbial killing and immune tolerance.

## 4. Copper-Mediated Inflammatory Signaling

### 4.1. Copper Homeostasis in Inflammation

Copper signaling is a critical driver of inflammation in macrophages; inflammatory stimuli raise intracellular copper and drive it into mitochondria across species. The elevation of intracellular copper results from the CD44–mitochondrial Cu (II) axis (Table 2). CD44 cytosolic tail directly binds CTR1, promoting its endocytosis and trafficking to the outer mitochondrial membrane. Mitochondrial CTR1 imports Cu^+^ into the matrix, thereby elevating local copper concentrations and enhancing CD44-mediated copper uptake along with mitochondrial copper accumulation [38,39]. Targeting the mitochondrial copper signaling axis with copper chelators like LCC-12 has been shown to mitigate inflammation in both in vitro and in vivo models, including reduced expression of inflammatory markers, reduced body temperature, and improved survival [39,40].

Copper plays a dual role in inflammation. On one hand, copper significantly upregulates phosphorylated p65 (p-p65) protein expression, promoting its nuclear translocation to bind promoter regions of pro-inflammatory cytokines (IL-1β, TNF-α, IL-6, iNOS, COX-2), thereby initiating mRNA transcription and enhancing cytokine release [38]. Copper enhances macrophage copper uptake through upregulated membrane glycoprotein CD44, simultaneously upregulating hyaluronan synthases (HAS1/2/3) and downregulating copper efflux proteins (ATP7A/B) [41]. This drives intracellular copper accumulation, forming a bioactive Cu^2+^ pool in mitochondria. Cu^2+^ catalyzes NADH oxidation by H_2_O_2_ to sustain the NAD(H) redox cycle, maintaining critical metabolites (α-ketoglutarate, acetyl-CoA) required for iron-dependent demethylases and acetyltransferases. These enzymes mediate epigenetic reprogramming (e.g., H3K27ac enrichment) to activate inflammatory gene expression [39]. On the other hand, copper chelators like LCC-12 selectively bind mitochondrial Cu^2+^, inhibiting its catalytic activity. This disrupts NAD(H) cycling, depletes NADH/NAD^+^ pools, and reduces α-ketoglutarate/acetyl-CoA synthesis, thereby suppressing epigenetic modifiers (e.g., HDACs, HATs) and reversing chromatin states to silence IL-6/TNF promoters. Mechanistically, LCC-12 attenuates macrophage/dendritic/T-cell activation (evidenced by CD86/CD80/CD40/CD83/CD25/CD69 downregulation) and inhibits epithelial–mesenchymal transition (EMT) in tumors. In in vivo models, including LPS-induced endotoxemia, CLP-induced sepsis, and SARS-CoV-2 infection, it can also reduce the expression of inflammatory markers (such as iNOS and interleukin-1 receptor-associated kinase 4), enhancing NF-κB signaling and promoting the production of pro-inflammatory cytokines. These positions copper as both a direct antimicrobial agent and a signaling molecule in innate immunity [39].

### 4.2. Copper Promotes Inflammation by Catalyzing the Redox Cycle of NAD(H)

Copper (II) in mitochondria catalyzes the redox cycle of NAD(H), leading to an increase in hydrogen peroxide in mitochondria by increasing the expression of superoxide dismutase 2 (SOD2) and decreasing catalase activity. Copper (II) acts as a catalyst to promote the reaction of NADH with hydrogen peroxide to produce NAD. Mitochondrial Cu^2+^ maintains the NAD^+^ pool and catalyzes the reaction between NADH and hydrogen peroxide (H_2_O_2_) to drive the NAD^+^/NADH redox cycle [42]. This process sustains the inflammatory microenvironment by maintaining critical metabolic intermediates such as α-ketoglutarate (αKG) and acetyl-CoA, which are essential for preserving the activity of iron-dependent demethylases and acetyltransferases. These enzymes mediate epigenetic reprogramming (e.g., H3K27ac enrichment) to activate pro-inflammatory gene expression, thereby establishing a metabolic-epigenetic axis essential for chronic inflammation [39]. High NADH initially blocks SIRT/AMPK and can transiently elevate acetylation, but its dominant late effect is to fuel HDAC3, G9a and Nuclear Receptor-Binding SET Domain Protein 2 (NSD2), replacing H3K36ac, H3K14ac and H3K9ac with H3K36me3 and H3K9me2. These repressive methyl-marks lock inflammatory loci in a silent state, translating metabolic redox status directly into transcriptional output, which is associated with downregulation of inflammatory gene expression [43].

### 4.3. Copper Participates in Inflammation by Activating ALPK1 Kinase

The innate immune system is the host’s first line of defense against pathogen invasion, and host cells sense pathogen-associated molecular patterns (PAMPs) through pattern recognition receptors (PRRs), trigger downstream signaling cascades, and ultimately induce the expression of various cytokines and antimicrobial peptides to defend against pathogens [44]. Copper ions play an important role in the fight against bacterial infections in host cells, and the mechanism is to enhance the host’s innate immune response by activating ALPK1 kinase. Copper ions can directly bind to ALPK1 and regulate its kinase activity, thereby enhancing the sensitivity of host cells to the bacterial metabolite ADP-heptose and promoting the activation of the NF-κB signaling pathway [29]. Copper ions can directly bind to ALPK1 and enhance its kinase activity, which in turn promotes the activation of the downstream NF-κB signaling pathway, leading to increased production of pro-inflammatory cytokines. Recent research has revealed that the essential trace element copper (Cu^+^/Cu^2+^) is not merely a bystander but a critical direct activator of this pathway, amplifying the inflammatory response to infection. In experiments involving titanium-based copper-containing ceramic coatings, it has been demonstrated that Cu^2+^ induces macrophage polarization toward the pro-inflammatory M1 phenotype, thereby activating inflammatory responses. In vitro and in vivo studies on Ti-Cu/SLA composite coatings have shown that Cu^+^/Cu^2+^ amplifies infection-associated inflammatory responses by activating the NF-κB signaling pathway and promoting reactive oxygen species (ROS) generation, thereby enhancing bacterial clearance capacity. In vivo experiments on 317L-Cu stainless steel have confirmed that Cu^2+^ actively enhances the protective inflammatory response at the local infection site, thereby preventing implant-related infections. Furthermore, studies on copper-containing mesoporous bioglass have demonstrated that Cu^2+^ regulates infection-related wound inflammation by activating macrophage inflammatory gene expression and ROS generation, effectively controlling bacterial proliferation while maintaining tissue repair mechanisms [45].

## 5. Copper in Tissue Repair and Regeneration

Beyond its immunomodulatory roles, copper plays a vital part in tissue repair [46]. Copper serves as a critical component of lysyloxidase (LOX), an enzyme that catalyzes the oxidative deamination of peptidyl lysine residues, initiating cross-linking reactions of lysine-derived aldehydes. This process promotes the maturation of collagen and elastin fibers during wound healing. Collagen and elastin are core structural proteins essential for skin tissue repair and maintaining structural integrity. By activating LOX enzymatic activity, copper facilitates the formation of covalent cross-links between these proteins, thereby enhancing their mechanical stability and tensile strength. This copper-dependent cross-linking mechanism not only strengthens the extracellular matrix but also ensures proper tissue remodeling and functional recovery in damaged tissues [47,48]. It regulates the synthesis of collagen and extracellular matrix components essential for wound healing. Copper supports the polarization of macrophages toward reparative M2 phenotypes, aiding tissue regeneration. Copper ions regulate the synthesis of collagen and other extracellular matrix components, which are essential for wound healing and tissue regeneration [49]. Additionally, copper-mediated metabolic reprogramming supports the transition from pro-inflammatory to reparative macrophage phenotypes, facilitating efficient tissue repair [50]. When applied topically, copper has been found to have low toxicity, making it a safe option for wound dressings and other medical applications. This is particularly important as it minimizes the risk of adverse reactions while providing therapeutic benefits [51]. Copper does not act on a single pathway—it simultaneously couples angiogenesis to hypoxia via HIF-1α, provides the enzymatic cofactor for ECM maturation (LOX), and tilts macrophages toward a reparative M2 state [52].

## 6. Effects of Copper Deficiency on Animal Health

Copper (Cu), an essential trace element in organisms, exerts a central regulatory effect on the growth and development, energy metabolism, redox homeostasis, and immune defense of both monogastric and ruminant animals. Copper deficiency is capable of inducing extensive systemic pathological alterations and significantly compromising animal production performance as well as disease resistance. In veterinary clinical settings, anemia represents one of the most typical pathological manifestations of copper deficiency. The primary mechanism underlying this condition involves the impairment of ceruloplasmin-mediated oxidation of Fe^2+^ to Fe^3+^, leading to disturbances in iron transport and hemoglobin biosynthesis. Notably, a state of functional iron deficiency may still ensue even when dietary iron intake is sufficient [53].

Copper deficiency exerts an equally significant impact on the skeletal and connective tissue systems. Given that the activity of lysyl oxidase is dependent on the presence of copper ions [54], copper deficiency can induce cross-linking disorders of collagen and elastin, which in turn leads to pathological alterations including skeletal dysplasia, joint abnormalities, and connective tissue fragility.

This effect is particularly prominent in rapidly growing juvenile animals, which is often accompanied by clinical signs such as rough, discolored, and brittle hair coats [53].

In addition, copper plays a crucial role in preserving the structural integrity of the cardiovascular system. Under conditions of copper deficiency, abnormal formation of elastic fibers increases vascular wall fragility and induces arterial dilatation as well as circulatory dysfunction [55].

Notably, copper deficiency is also closely linked to reproductive disorders, as it interferes with key reproductive processes including follicular development, embryo implantation, and spermatogenesis. These disruptions subsequently result in decreased conception rates, elevated embryonic mortality, and impaired overall reproductive performance.

It is noteworthy that there are significant species differences in copper metabolism between ruminants and monogastric animals. Due to their complex rumen microbial system, copper absorption in ruminants is more susceptible to interference from antagonistic elements such as molybdenum, sulfur, and iron [56]. Particularly under conditions of high molybdenum and high sulfur in pasture, insoluble copper thiomolybdate complexes can form, significantly reducing copper bioavailability. Consequently, animals may still exhibit secondary copper deficiency even when the total dietary copper level is normal. Therefore, ruminants such as cattle and sheep are more prone to chronic copper deficiency syndrome, clinically manifested as growth retardation, coat discoloration, skeletal abnormalities, and neurological damage, among other symptoms [57]. Lamb “swayback” is a typical copper-deficiency-related neuropathy [56]. In contrast, monogastric animals have higher copper absorption efficiency, and some species (e.g., pigs) exhibit strong tolerance to high-copper diets; hence, copper is often used as a growth-promoting additive in livestock production. However, long-term high copper intake may also induce hepatic accumulation and oxidative stress damage, indicating that copper homeostasis imbalance encompasses not only deficiency but also potential toxicity risks.

## 7. Copper Content in Major Feed Ingredients and Strategies to Alleviate Dietary Copper Deficiency

### 7.1. Copper Content and Bioavailability in Major Feed Ingredients

The copper content in natural feed ingredients exhibits significant variation. Generally, cereal-based feeds are relatively low in copper, while the copper levels in forages and legumes are highly dependent on soil mineral composition [58]. In pig production systems, the copper content of basal diets can range widely from 38 mg/kg to 188 mg/kg dry matter, reflecting both the inherent differences in raw materials and the impact of supplemental additions [59]. Crucially, the total copper content in feed does not equate to the amount biologically available to the animal. The bioavailability of copper is significantly influenced by interactions with other minerals. A primary antagonistic interaction involves molybdenum (Mo) and sulfur (S). When diets contain more than 5.0 mg/kg Mo and above 0.33% S, the copper absorption efficiency in ruminants is markedly reduced [60]. This interaction can induce a state of secondary copper deficiency even when total dietary copper levels appear adequate, as the formation of insoluble copper thiomolybdate complexes in the digestive tract renders the copper unavailable for absorption [58,60].

### 7.2. Integrated Strategies to Address Dietary Copper Deficiency

Addressing copper deficiency requires a multi-faceted approach beyond simple supplementation.

Precision Copper Supplementation: For animals diagnosed with deficiency, direct copper supplementation is an effective intervention. Oral administration of copper sulfate solution has proven effective in completely curing copper deficiency and restoring normal copper status in ruminants [61].

Feed Formulation Optimization and Novel Additives: Optimizing diet formulation is fundamental, requiring careful control of antagonistic element levels (e.g., Mo < 5.0 mg/kg, S < 0.33%) [60]. Novel mineral additives show promise. Replacing inorganic copper and zinc with nanoscale copper–zinc alloys in poultry diets has demonstrated positive production effects, including increased serum protein and improved liver enzyme activity, without inducing oxidative stress. This nanoscale form demonstrated a cumulative effect, increasing copper content in the liver by 36.5% and in feathers by 2.5 times, indicating superior bioavailability and retention compared to traditional inorganic forms [62].

Agronomic Biofortification: This sustainable strategy addresses deficiency at the source. Foliar application of copper fertilizers can significantly increase the copper content of pastures and forage crops, providing a rapid and economical method for immediate enrichment and reducing the need for high levels of feed additives [58].

Scientific Feed and Manure Management: Feed management is the primary lever for controlling copper entry into the environment. Dietary copper content and form directly influence the copper concentration in manure, which can double on a dry matter basis after treatment processes like anaerobic digestion [59]. Therefore, adjusting feed strategies based on the entire “feed-animal-manure-treatment” chain is essential to meet animal nutritional requirements while producing compliant organic fertilizers.

In conclusion, managing copper nutrition is a systemic challenge. Effective solutions combine precision supplementation, dietary formulation that accounts for mineral interactions, the adoption of high-bioavailability copper sources like nanoscale alloys, and sustainable practices like biofortification, all within a framework of responsible nutrient management.

## 8. Conclusions and Future Perspectives

**Figure 1:**
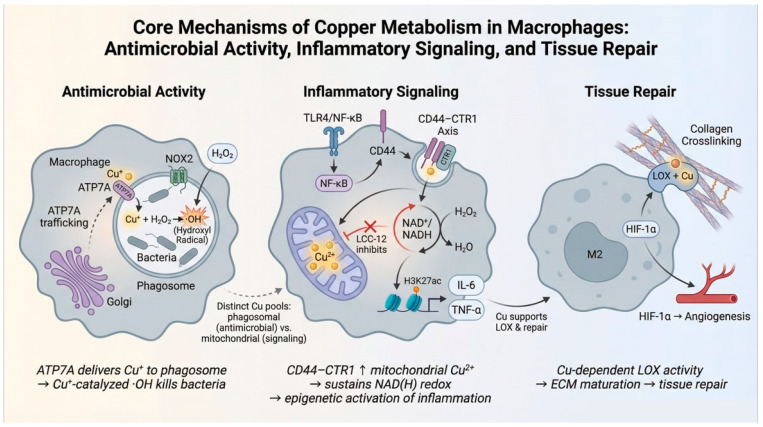
Copper metabolic mechanisms in macrophages. On the left is copper’s antimicrobial activity: macrophages eliminate pathogens through a synergistic mechanism of copper transport and reactive oxygen species-mediated killing. In the middle is inflammatory signal transduction: copper drives the expression of pro-inflammatory factors and the maintenance of inflammatory responses by regulating the mitochondrial redox state and epigenetic modifications. On the right is tissue repair: copper promotes the maturation of the extracellular matrix and angiogenesis by activating key enzymes such as lysyl oxidase, thereby achieving tissue repair.

Great progress has been made in the study of the transport, characteristics and functions of macrophages in physiology and pathology in recent years. One of the most deeply studied aspects of macrophage biology is its role in phagocytosis in the body’s immunity [63]. These cells can recognize and ingest pathogens, initiating signaling pathways that produce inflammatory mediators that work together to eliminate pathogens and infected cells [64]. The mechanism of copper metabolism in macrophages is shown in Figure 1. However, the molecular mechanism behind the inflammatory response is not fully understood. With the development of immunotherapy for the treatment of inflammatory diseases, macrophages have become an important component of the immune system; their role and potential in immunotherapy have accordingly received more attention [65]. How to enhance the effect of immunotherapy by regulating the function of macrophages has become a new hot field; a lot of basic research and clinical treatments are aimed at macrophage markers and their related signaling pathways [66].

Furthermore, mechanisms such as determining whether copper is elevated in phagosomes in which bacterial pathogens reside in macrophages and the genetic phenotype of the copper detoxification system in various infection models remain to be further explored [34]. Therefore, the potential risks and benefits of copper need to be carefully assessed when considering its use as a treatment. Future research needs to further explore the specific mechanisms of copper ions in macrophage function and develop novel anti-infection strategies based on copper ion regulation. In particular, future studies should investigate the different mechanisms of action of copper ions in M1 and M2 macrophages, and their potential applications in chronic inflammation and tissue repair [67,68]. In-depth studies are needed on the specific mechanisms of copper ions in the antimicrobial activity of macrophages, including how they enhance bactericidal ability by catalyzing the production of hydroxyl radicals, and the differences in the role of copper ions in different pathogen infections [69,70].

## Figures and Tables

**Table 1:** Antibacterial mechanisms of copper in macrophages.

Experimental Model/Cells	Copper Resource	Target	Bacterial Species	Ref.
RAW264.7 macrophages + LPS/IFN-γ	Endogenous copper uptake via CTR1 and transport by ATP7A	CTR1/ATP7A pathway; phagolysosomal copper loading; ROS/Fenton reaction enhancement	*Escherichia coli* infection	HODGKINSON V et al. 2012 [33]
ATP7A-silenced RAW264.7 macrophages	Intracellular copper transported by ATP7A	ATP7A-dependent phagosomal bactericidal activity	*Escherichia coli* infection	
Activated macrophages/Mtb-infected macrophages	ATP7A-mediated copper transport to phagosome	Phagosomal Cu overload → ROS burst, membrane damage, enzyme inactivation	*Mycobacterium tuberculosis*	HU D et al. 2025 [34]
Mtb-infected macrophages (bacterial Cu detox response)	Host-derived intracellular Cu stress	MmcO-mediated Cu detoxification → ROS resistance and survival	*M. tuberculosis*	

**Table 2:** Polarization effects of copper in macrophages.

Experimental Model/Cells	Copper Resource	Polarization Type	Target	Ref.
Bovine macrophages + LPS	Endogenous copper uptake induced by LPS	M0 → M1 polarization	Increased IL-1β and IL-6 expression	GUO H. et al. 2024 [38]
Bovine macrophages + LPS + CuSO_4_ (25 μM)	Exogenous CuSO_4_ supplementation	Enhanced M1 polarization	NF-κB activation; p65 phosphorylation	
Bovine macrophages + LPS + CuSO_4_ (50 μM)	High-dose exogenous CuSO_4_	Enhanced M1 polarization	Increased TNF-α, IL-1β, IL-6, iNOS, and COX-2	
Bone marrow-derived macrophages	CD44-mediated uptake of extracellular Cu^2+^	M0 → M1-like inflammatory macrophages	CD44-dependent copper uptake; reactive mitochondrial Cu(II); NAD(H) redox regulation	SOLIER S et al. 2024 [39]

## Data Availability

No new data were created or analyzed in this study.

## Abbreviations

ATOX1	Antioxidant Protein 1
CCO	Cytochrome c oxidase
CLP	Cecal ligation and puncture
COX-2	Cyclooxygenase-2
CP	Ceruloplasmin
CTR1	Copper Transport Protein 1
DAG	Diacylglycerol
G9a	Euchromatic histone lysine methyltransferase 2 (EHMT2)
GBP	Guanylate-binding proteins
HDAC3	Histone deacetylase 3
H3K36ac	Histone H3 lysine 36 acetylation
H3K36me3	Histone H3 lysine 36 trimethylation
H3K9ac	Histone H3 lysine 9 acetylation
H3K9me2	Histone H3 lysine 9 dimethylation
LDs	Lipid droplets
LOX	Lysyl oxidase
mROS	Mitochondrial reactive oxygen species
NO	Nitric oxide
NSD2	Nuclear receptor-binding SET domain protein 2
AMPs	Pathogen-associated molecular patterns
PPP	Pentose phosphate pathway
PRRs	Pattern recognition receptors
RNAi	RNA interference
ROS/RNS	Reactive oxygen/nitrogen species
SOD	Superoxide dismutase
TCA	Tricarboxylic acid cycle
TLRs	Toll-like receptors
